# Molecular characterization of breast tumor cells in sentinel lymph nodes

**DOI:** 10.1186/1753-6561-7-S2-P16

**Published:** 2013-04-04

**Authors:** Clarissa Torresan, Savana CL Santos, Silma F Pereira, Marcia M Oliveira, Rubens S Lima, Cícero Urban, Raquel Wall, Jordi Camps, Bassem R Haddad, Enilze MSF Ribeiro, Iglenir J Cavalli, Luciane R Cavalli

**Affiliations:** 1 Department of Genetics, Federal University of Parana, Curitiba, PR, Brazil; 2 Lombardi Comprehensive Cancer Center, Georgetown University, Washington DC, USA; 3 Department of Oncology, Nossa Senhora das Graças Hospital, Curitiba, PR, Brazil; 4 Departament of Patology, Clinical Hospital of UFPR, Curitiba, PR, Brazil; 5 Section of Cancer Genomics, Genetics Branch, NIH, Bethesda, MD, USA

## Background

The sentinel lymph node (SLN) is the first node in the axilla to harbor malignant cells in breast tumors with metastasis, and its positivity is an indication for axillary lymph node dissection (ALND). The accurate evaluation of the SLN, to identify even the smallest metastatic foci is essential prior to complete ALND. We propose to characterize the tumor cells present at the SLN using global genomic approaches to identify DNA copy number changes.

## Materials and methods

Paired cases of primary breast tumors (PBT) and SLN were analyzed using Comparative Genomic Hybridization CGH (30 pairs) and array-CGH (10 pairs). The formalin-fixed paraffin-embedded samples were microdissected and hybridized with normal DNA to metaphase chromosomes and to a 44k oligo-array platform. Validation of array-CGH was performed by FISH and Taqman CopyNumber Assay.

## Results

A remarkable similarity in the DNA profiles between the SLN metastatic lesions and the corresponding PBT, despite of the high level of heterogeneity, was observed among the cases. Gains/amplifications of chromosomal regions (mainly in 1p, 1q, 6p, 11p, 16, 17, 18q, 19, 20, 21 e 22) were most frequently observed than losses/deletions (mainly in 1p, 2q, 4q, 6q, 12q, 13q, 18q e Xq). Gene annotation analysis of these regions revealed the presence of several genes with relevance to the axillary lymph node metastatic process, involving critical cellular pathways, such as apoptosis, DNA repair and cellular proliferation.

## Conclusions

The assessment of copy number changes in the SLN metastasis using genomic profiling is a very sensitive method and will lead to the identification of genomic alterations that can reliably characterize the breast tumor cells present in this node. Once these alterations are validated, they can be used as *molecular markers*, to identify patients at higher risk of developing axillary lymph node metastasis.

## Financial support

UFPR, CAPES and CNPq.

